# Supplementary material for: Manipulation of the human tRNA pool reveals distinct tRNA sets that act in cellular proliferation or cell cycle arrest
Source: eLife. 2020 Dec 24;9:e58461. doi: 10.7554/eLife.58461 (PMC7781600; doi:10.7554/eLife.58461)
Supplement: Transparent reporting form [file elife-58461-transrepform.docx]

***eLife’s* transparent reporting form**

We encourage authors to provide detailed information *within their submission* to facilitate the interpretation and replication of experiments. Authors can upload supporting documentation to indicate the use of appropriate reporting guidelines for health-related research (see [EQUATOR Network](http://www.equator-network.org/%20)), life science research (see the [BioSharing Information Resource](https://biosharing.org/)), or the [ARRIVE guidelines](http://www.plosbiology.org/article/info:doi/10.1371/journal.pbio.1000412) for reporting work involving animal research. Where applicable, authors should refer to any relevant reporting standards documents in this form.

If you have any questions, please consult our Journal Policies and/or contact us: [editorial@elifesciences.org](mailto:editorial@elifesciences.org).

**Sample-size estimation**

- You should state whether an appropriate sample size was computed when the study was being designed
- You should state the statistical method of sample size computation and any required assumptions
- If no explicit power analysis was used, you should describe how you decided what sample (replicate) size (number) to use

Please outline where this information can be found within the submission (e.g., sections or figure legends), or explain why this information doesn’t apply to your submission:

For CRISPR-targeted tRNA variants experiments (Figures 3-5), sgRNA library was transduced at a Multiplicity of Infection (MOI) of 0.3, aiming for coverage of, on average, 30,000-60,000 cells per sgRNA (dependent on the cell line). In figure 5 the transduced cell populations were FACS-sorted when the top and bottom 5% of the population were collected (minimum 10,000 cells per bin). This information can be found in the Materials and Methods section.

**Replicates**

- You should report how often each experiment was performed
- You should include a definition of biological versus technical replication
- The data obtained should be provided and sufficient information should be provided to indicate the number of independent biological and/or technical replicates
- If you encountered any outliers, you should describe how these were handled
- Criteria for exclusion/inclusion of data should be clearly stated
- High-throughput sequence data should be uploaded before submission, with a private link for reviewers provided (these are available from both GEO and ArrayExpress)

Please outline where this information can be found within the submission (e.g., sections or figure legends), or explain why this information doesn’t apply to your submission:

Each experiment includes two biological repeats (described in figure legends), meaning two cell populations that were transduced with a pooled sgRNA-viral particle, independently and separately. Upon acceptance of the manuscript, the sequencing data will be available at public database.

**Statistical reporting**

- Statistical analysis methods should be described and justified
- Raw data should be presented in figures whenever informative to do so (typically when N per group is less than 10)
- For each experiment, you should identify the statistical tests used, exact values of N, definitions of center, methods of multiple test correction, and dispersion and precision measures (e.g., mean, median, SD, SEM, confidence intervals; and, for the major substantive results, a measure of effect size (e.g., Pearson's r, Cohen's d)
- Report exact p-values wherever possible alongside the summary statistics and 95% confidence intervals. These should be reported for all key questions and not only when the p-value is less than 0.05.

Please outline where this information can be found within the submission (e.g., sections or figure legends), or explain why this information doesn’t apply to your submission:

The data shown in all heatmaps and violin plots are presented as mean values of the two biological repeats, for each experiment and each time point. The central mark of the box plot in figure 2B indicates the median, and the bottom and top edges of the box indicate the 25th and 75th percentiles, respectively. The bar plot in figure 2C presents the mean ± SE.

Comparisons between tRNA groups shown in violin plots were tested using Wilcoxon rank sum test. Comparisons between tRNA levels in different time points (Figure 2C) were tested using two- sample t-test. The p-values can be found both in the Results section and in the figure legends.

Linear correlations were tested using Pearson correlation. The correlation coefficient r and p-values can be found both in the Results section and in the figure legends.

(For large datasets, or papers with a very large number of statistical tests, you may upload a single table file with tests, Ns, etc., with reference to sections in the manuscript.)

**Group allocation**

- Indicate how samples were allocated into experimental groups (in the case of clinical studies, please specify allocation to treatment method); if randomization was used, please also state if restricted randomization was applied
- Indicate if masking was used during group allocation, data collection and/or data analysis

Please outline where this information can be found within the submission (e.g., sections or figure legends), or explain why this information doesn’t apply to your submission:

**Additional data files (“source data”)**

- We encourage you to upload relevant additional data files, such as numerical data that are represented as a graph in a figure, or as a summary table
- Where provided, these should be in the most useful format, and they can be uploaded as “Source data” files linked to a main figure or table
- Include model definition files including the full list of parameters used
- Include code used for data analysis (e.g., R, MatLab)
- Avoid stating that data files are “available upon request”

Please indicate the figures or tables for which source data files have been provided:

Upon acceptance of the manuscript, the raw data tables and Matlab codes will be available
